# Differences in on-ground and aloft conditions explain seasonally different migration paths in Demoiselle crane

**DOI:** 10.1186/s40462-022-00302-z

**Published:** 2022-01-31

**Authors:** Batbayar Galtbalt, Nyambayar Batbayar, Tuvshintugs Sukhbaatar, Bernd Vorneweg, Georg Heine, Uschi Müller, Martin Wikelski, Marcel Klaassen

**Affiliations:** 1 Centre for Integrative Ecology, School of Life and Environmental Science, Deakin University, Victoria, Australia; 2 Wildlife Science and Conservation Center of Mongolia, Ulaanbaatar, Mongolia; 3 Department of Migration, Max Planck Institute of Animal Behavior, Radolfzell, Germany

**Keywords:** Loop migration, Environmental conditions, Wind support, Thermals, Temperature, NDVI, *Anthropoides virgo*

## Abstract

**Background:**

Although some migratory birds may take different routes during their outbound and inbound migration, the factors causing these differential migrations to and from the breeding grounds, have rarely been investigated. In Northeast Asia, Demoiselle crane (*Anthropoides virgo*) performs one of the most extreme “loop” migrations known to date. During outbound migration, they cross the Himalayas to non-breeding sites in northwest India. Contrastingly, during inbound migration to the breeding grounds, they fly around the western end of the Himalayas. We hypothesise that differences in prevailing environmental conditions aloft and/or on-ground during both seasonal migrations are at the core of this phenomenon.

**Methods:**

Based on the tracking data of 16 individuals of tagged Demoiselle crane, we compared conditions during actual migration with those of simulated “reverse” migration (i.e. by adding 180 degrees to the flight direction and adding and subtracting half a year to the timestamps of outbound and inbound migration, respectively).

**Results:**

The comparison of actual and simulated “reverse” migration indicated that cranes would have encountered poorer aloft (wind support and thermal uplift) and on-ground conditions (temperature) if they had migrated in a reverse outbound migration and poorer on-ground conditions (Normalised Difference Vegetation Indexes [NDVI]) if they had migrated in a reverse inbound direction.

**Conclusions:**

Our analyses suggest that both on-ground and aloft conditions play a key role in explaining Demoiselle cranes’ loop migration, during the periods that they chose to use these alternative routes. Knowledge on the determinants of (differential) migration routes allow predicting migration decisions and may be critical in mitigating global change effects on animal migrations.

**Supplementary Information:**

The online version contains supplementary material available at 10.1186/s40462-022-00302-z.

## Background

It is well established that some migratory animals may follow widely different routes during outbound and inbound migration (i.e. migration from and to their breeding grounds, respectively), also known as “loop” migration [1]. The use of different routes may be age-dependent and occur only once during a life cycle, such as in Sharp-tailed sandpipers (*Calidris acuminata*) that breed in Arctic Siberia and spend the non-breeding season in Australasia. Whereas the adults follow the East Asian coast during their biannual migrations, juveniles often fly across the Bearing Strait to Alaska and then on to Australasia [2]. It may also be an annually recurring phenomenon for all ages, such as in American golden plover (*Pluvialis dominica*) [3] and Red-backed shrike (*Lanius collurio*) [4]. The hypothesised reasons for these differential migration routes are thought to be related to their consequences for the migrants’ time and energy budgets as well as their safety [1, 5], i.e. the three key factors that are thought to have shaped animal migratory behaviour [6].

Differential seasonal migration routes have been predominantly documented in avian species. Few of these studies profoundly researched the underlying causes for this phenomenon and generally assumed that it is an adaptation to differential seasonal variations in prevailing environmental condition along the two alternative routes [4, 7–14]. Klaassen et al. [9] tracked 14 Marsh harriers (*Circus aeruginosus*) to investigate the effect of the availability of suitable habitat and prevailing wind conditions on their migration path. They found that only wind was able to explain the Marsh harriers’ somewhat more easterly migration route between North-western Europe and Western Africa during outbound compared to inbound migration. Using on-the-ground observations, Sorte et al. [8] found that during inbound spring migration, 26 terrestrial bird species in the Western Flyway of North America followed a lower-elevation, less-direct route because of locally higher ecological productivity during that time of year. On the other hand, in the Eastern Flyway of North America, many birds undertake their outbound migration to the east of their inbound migration to make use of prevailing wind conditions over the Atlantic Ocean [1, 15, 16]. Also for birds in the Afro-Palearctic flyway, many examples of seasonally differential migratory routes have been documented, including Eleanor’s falcon (*Falco eleanorae*) [10], Red-backed shrike (*Lanius collurio*) [4], Common cuckoo (*Cuculus canorus*) [17], Barn swallow (*Hirundo rustica*) [7, 18], Northern wheatear (*Oenanthe oenanthe*) [11] and Nightjar (*Caprimulgus europaeus*) [19]. Consequently, researchers have argued that loop migration might well be the predominant strategy in this flyway [4, 19]. In addition to these examples of terrestrial bird species, several marine birds including Manx shearwater (*Puffinus puffinus*) in the Atlantic [20], and Sooty shearwater (*Puffinus griseus*) in the Pacific Oceans [13] have been found to undertake loop migrations, which have been suggested to be driven by prevailing large-scale wind patterns [21]. In all cases, it has been assumed that variations in environmental conditions along the alternative migratory tracks, both aloft (during migratory flight) and on the ground (during migratory staging), are the core determinants for the choice of the specific migratory route.

**Fig. 1:**
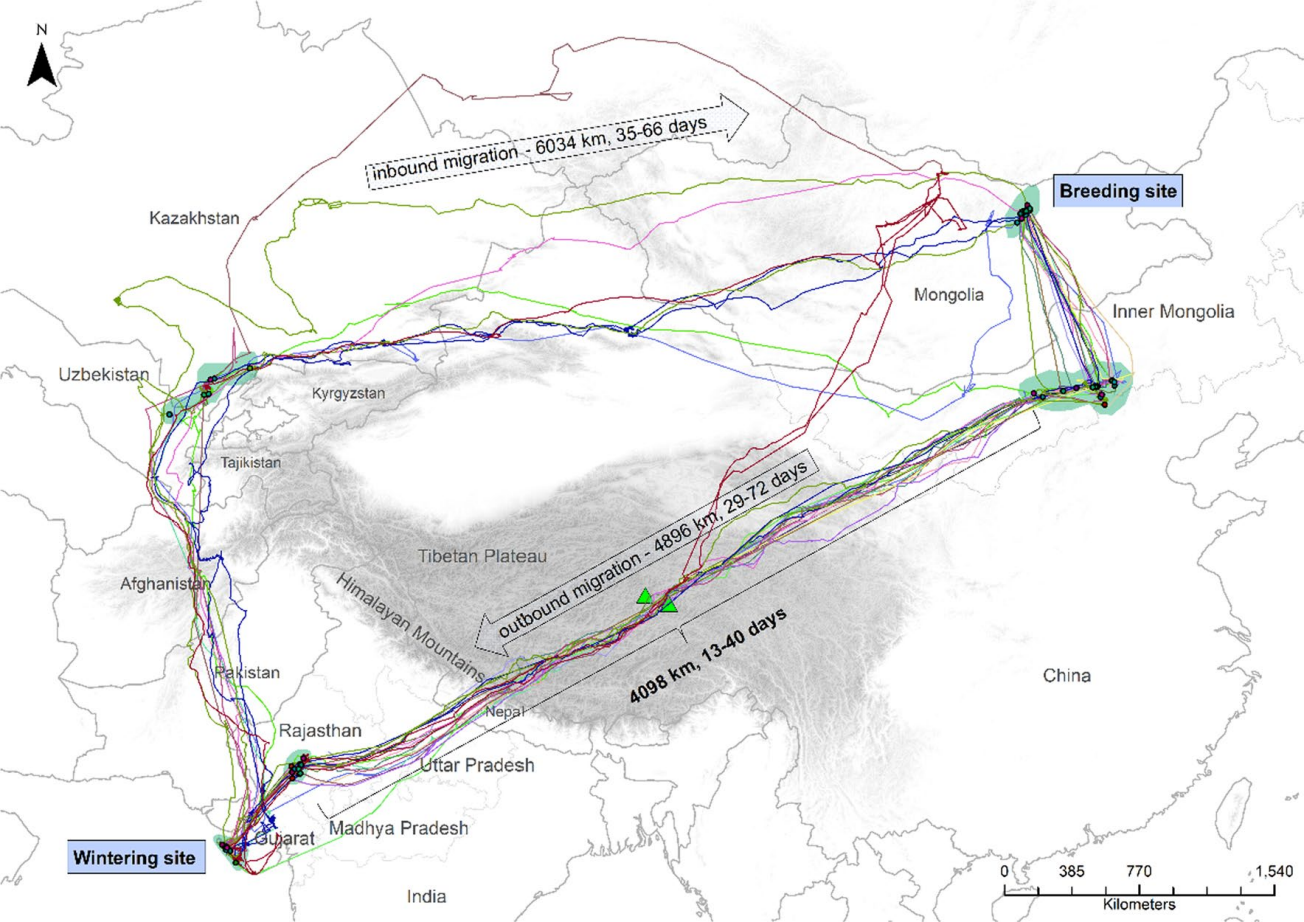
Migratory route and key staging areas of 16 satellite-tracked Demoiselle cranes. Green triangles indicate two mountain ranges on the Tibetan Plateau, i.e. Gar Kangri and Medu-Kun, through which the cranes migrated. Green areas indicate commonly used staging sites, and grey shading elevations in excess of 2000 m above mean sea level. Map was produced using ArcGIS v 10.5, using WGS 1984 World Mercator projection

Compared to all the above examples of loop migration, Demoiselle cranes (*Anthropoides virgo*) from the eastern parts of their distribution range in East Asia, have an exceptionally pronounced loop migration using dramatically different migratory routes during outbound and inbound migration. They migrate across the Himalayas during their outbound migration, but rather than flying back along the same route from their wintering grounds in Gujarat, India, they instead make a loop migration around the western end of the Himalayas [22, 23] and next fly in easterly direction, 1700 to 2650 km to the north of their outbound migratory track (Fig. 1). The reason for these vastly different seasonal migration routes is unknown and has not been investigated previously.

To add to the limited knowledge base on the drivers for loop migrations we aimed at identifying these for the extreme loop migration of Demoiselle cranes in East Asia. Hypothesising that also in this case variations in environmental conditions during the two migratory seasons may drive the preference for the two routes, we investigated temporal variations in environmental conditions and how these might affect migratory behaviour. To this end, using GPS-GSM tracking along with meteorological and Normalised Differential Vegetation Index (NDVI) data, we analysed the suitability of the environmental conditions on the ground for foraging and aloft for migratory flight along both outbound and inbound routes. We conducted these analyses at the time of their respective migrations along both trajectories, but also for a simulated, reverse-loop migration, reversing the birds’ flight direction and changing the timestamp by approximately half a year. Since wind conditions may importantly impact the energy and time costs of avian migration [24, 25], and also thermal uplift may play a role, notably in species such as cranes that may also engage in soaring flights during migration [26, 27], we considered both wind support and thermal uplift at flight height as potentially critical conditions aloft for migration. For on-ground, migratory-staging conditions we considered surface air temperature and plant biomass (i.e. NDVI) as proxy measures of foraging conditions. During migration Demoiselle cranes are known to forage mainly on crops and insects [28, 29], of which the availability is highly correlated with surface temperature [30, 31] and NDVI [32].

However, in so comparing conditions aloft and on-ground during actual and reverse migration, we did not account for the possibility that migrants may make use of short-term temporal variations in environmental conditions and notably wind conditions [33, 34]. Many migrants have been shown to pass through staging areas in peaks rather than at a continuous rate, occasionally delaying migration to wait for more favourable migratory conditions (e.g.* Cygnus bewickii*; [35, 36]). As a consequence, directly comparing the favourability of actual migratory conditions during one season with time-shifted (i.e. by half a year) conditions during the alternative season (i.e. reverse-simulated migration) would ignore the possibility of such fine-tuning on the actual migratory conditions. To investigate the potential bias in our comparison of actual with reverse-simulated migrations due to the possible existence of migratory fine-tuning, we also studied the effects of relatively small time-shifts (i.e. days to weeks) on migratory conditions aloft. We conducted this for aloft and not for on-ground conditions since conditions aloft are more variable from day to day than on-ground conditions.

## Methods

### Tracking and environmental data acquisition

We caught nine adult and eighteen juvenile Demoiselle cranes at their breeding ground in Northeast Mongolia (N48.3°; E110.3°) in both 2016 and 2019. The adults were caught using leg snare traps between 15 and 25 June whereas the juveniles were caught by hand shortly before fledging between 5 and 7 August. We deployed 15 g, solar-powered GPS/GSM transmitters (produced by Max-Planck Institute of Animal Behavior-University of Konstanz, Germany) on the right tibiotarsus of each individual using the method described in Ellis et al. [37].The transmitters were programmed to record positions at 20 min intervals and had a geographic positioning accuracy of ± 10 m while altitude was measured with an accuracy of ± 25 m.

Data from 16 out of the 27 transmitters were included in the analysis as data from eleven transmitters logged very few points or discontinued functioning before migration completion. Excluding the breeding (> 48°N; > 110°E) and wintering grounds (< 27°N; < 75°E), the transmitters produced 61,136 migration data points, which were classified as either “in-flight” or “stationary” for the analysis of migration in relation to conditions aloft and on-ground, respectively. We classified the fix as in-flight if ground speed (instantaneous measurements provided by the transmitter) was greater than 3 m/s and step length (distance between consecutive fixes) was above 5 km, and stationary if ground speed was less than 1 m/s. We omitted 5143 data points that did not meet any of these criteria. Stopover sites were defined as sites where birds spend more than two days within a radius of ten kilometres. Any in-flight fixes during a stopover were removed from the data set. Fixes outside the geographic breeding and wintering grounds as defined above and within the periods September to December and March to May were assigned as representing outbound (autumn; 2315 in-flight and 19,349 stationary fixes comprised of 21 migration events of 16 individuals) and inbound (spring; 2818 in-flight and 18,813 stationary fixes comprised of nine migration events of seven individuals) migration, respectively.

We annotated each in-flight fix with conditions aloft (i.e. wind support and thermal uplift) and each stationary fix with on-ground conditions (i.e. ambient temperature at 2 m above ground level and Normalised-Difference Vegetation Index, NDVI). The spatially and temporally specific meteorological variables used for calculating wind support and thermal uplift were obtained from the European Centre for Medium-Range Weather Forecast’s (ECMWF) ERA-5 hourly dataset for 18 pressure levels between 400 to 1000 hectopascal, via the Copernicus Climate Data Store [38]. Ambient temperature and NDVI were obtained via the Env-DATA system, which also uses data from ERA-5 and the National Aeronautics and Space Administration (NASA) as its primary data source for former and latter respectively [39]. The ERA-5 dataset has a spatial resolution of 0.25 degrees and a vertical resolution of 250–500 m, while for NDVI the spatial resolution was 250 m with a temporal resolution of 16 days. To obtain the most probable NDVI estimate at each fix we used bilinear spatio-temporal interpolation available within the Env-DATA system. The extracted wind data at the bird’s flight height, west to east (zonal, U_z_, m/s) and south to north (meridional, V_m_, m/s) wind components, together with flight direction (i.e. the direction in which the bird flies, θ_f_, which was estimated based on consecutive fixes using the ‘angle’ function within R-package move [40]) were used to calculate wind support (WS, m/s) following Safi et al. [41], using:

$$WS=\sqrt{{{V}_{m}}^{2}+{{U}_{z}}^{2}}\times \mathrm{cos}\left(\mathrm{atan}2({V}_{m},{U}_{z})-\frac{{2\pi \theta }_{f}}{360}\right)$$

We estimated thermal uplift following Bohrer et al. [42], using:

$${\omega }^{*} ={[gzH/T]}^{1/3}$$

where *g*-gravitational acceleration, *z* is boundary layer height, *H* is the surface sensible heat flux and *T* is potential temperature.

### Simulation of reverse and time-shifted migration

Using the recorded migration tracks and both in-flight and on-ground fixes, we simulated reverse migration using a seasonal time-shift to evaluate what atmospheric and on-ground conditions cranes would likely encounter if they would fly north using the outbound migratory path and south using the inbound migratory path. The process (graphically outlined in Fig. S1) started with reversing the time stamps of each individual’s outbound and inbound migration track. Next, we added 180 degrees to the birds’ flight directions (since flight directions are used for calculation of wind support). Finally, we added 195 days to the timestamps of outbound and subtracted 195 days from the timestamps of inbound migrations. These timestamp modifications were based on the median departure and arrival dates of September 6th and October 22nd for outbound and March 25th and May 08th for inbound migration, respectively. The simulated migration data were subsequently annotated with environmental condition data as described above.

To investigate the potential bias in our comparison of actual with reverse-simulated migrations due to the possible existence of migratory fine-tuning, we studied the effects of fixed time-shifts on migratory conditions aloft. Using all in-flight fixes of the original tracks, we systematically changed the timestamp by adding or subtracting either one to seven days, two weeks, or a one-month time period, resulting in a total of 18 different time-shift scenarios [43]. These time-shifted data sets were subsequently annotated with wind and thermal uplift following the same procedures as outlined above and compared with the annotated data of the original tracks.

### Analysis

All calculations and statistical analyses were conducted in R v. 4.1.1 [44]. To evaluate whether migratory conditions aloft and/or on-ground can explain the cranes outspoken loop-migration, we tested for differences in environmental conditions between actual and simulated reverse migration using linear mixed effect models. A total of eight tests were conducted in which each of the four environmental variables during both outbound and inbound migration were used as a response variable, and data type (either actual or simulated) was entered as a fixed effect and individual crane as a random effect explanatory variable. In the analyses we allowed for both a random intercept as well as a random slope to account for individual variation. For the four tests involving wind support and thermal uplift we used in-flight fixes only, while for the four tests involving the on-ground environmental variables temperature and NDVI we used stationary fixes only.

We tested if cranes are potentially fine-tuning their timing of migration to conditions aloft (wind support and thermal uplift) for outbound and inbound migration separately, yielding a total of four linear mixed effect models. In each of these models, using an in-flight data set combining the conditions aloft during actual migration as well as all 18 time-shifted scenario’s we used time-shift (ranging from 0 days to 1 month) as a (categorical) fixed effect and fix-ID nested within individual crane as random effect explanatory variables. Subsequent to each linear mixed effect model, to evaluate whether or not conditions were on average more favourable during actual than time-shifted migrations, we ran multiple comparison tests between the zero time-shift (actual) and all other time-shifts using the glht function and Dunnett contrasts [45] in package multcomp in R.

## Results

Environmental conditions aloft and on the ground varied greatly along both outbound and inbound migratory routes (Additional file 1: Table S1). As for the conditions aloft, wind support during inbound was significantly higher than outbound migration, while thermal uplift was similar (wind support: *t* test = 16.7, *df* = 4663, *p* < 0.001; thermal uplift: *t* test = − 0.09, *df* = 4,083, *p* > 0.05). On the other hand, on-ground conditions differed significantly between outbound and inbound migrations, with temperature being higher during outbound (*t *test = − 14.07, *df* = 37,996, *p* < 0.001) and NDVI being higher during inbound migration (*t* test = 11.5, *df* = 36,468, *p* < 0.001). We provided detailed summaries of migration phenology and en-route environmental conditions in the Additional file 1.

### Actual and simulated “reverse” migratory routes in relation to environmental condition

**Fig. 2:**
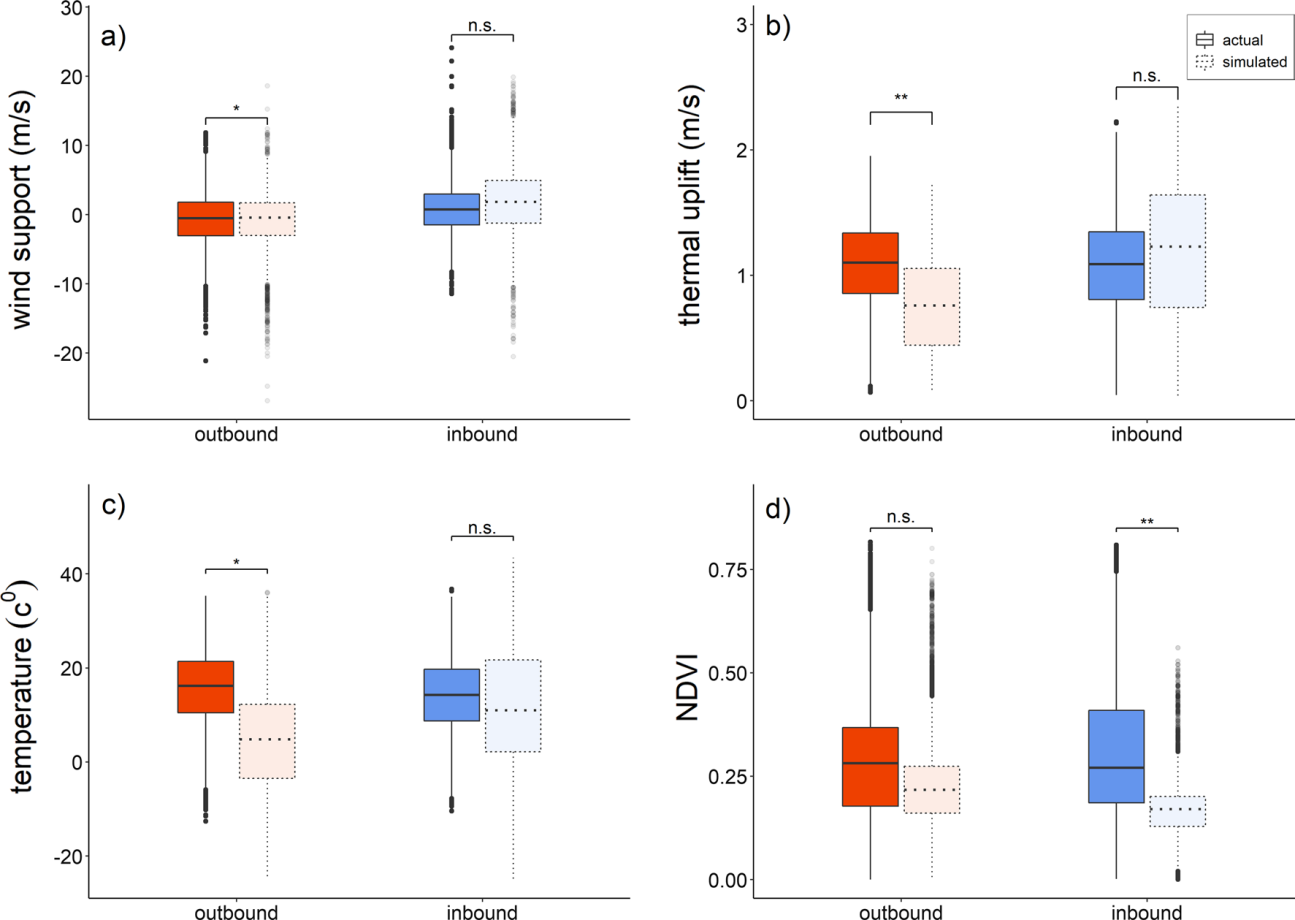
Aloft (wind support **a**, thermal uplift **b**) and on-ground conditions (surface temperature **c**, NDVI **d**) during actual (solid line) and simulated-reverse (dotted line) migration of Demoiselle crane. Boxplot shows the median (bar), upper and lower 25% quantiles (box), range (whiskers) and outliers (dots, i.e. > 1.5 times the interquartile range). Significance levels for comparisons across groups comparing outbound (red) with inbound (blue) and actual versus simulated data (transparent boxes) are indicated as *(*p* < 0.05), **(*p* < 0.01), ***(*p* < 0.001) and n.s. for non-significant. Statistics and sample sizes are presented in Table 1

**Fig. 3:**
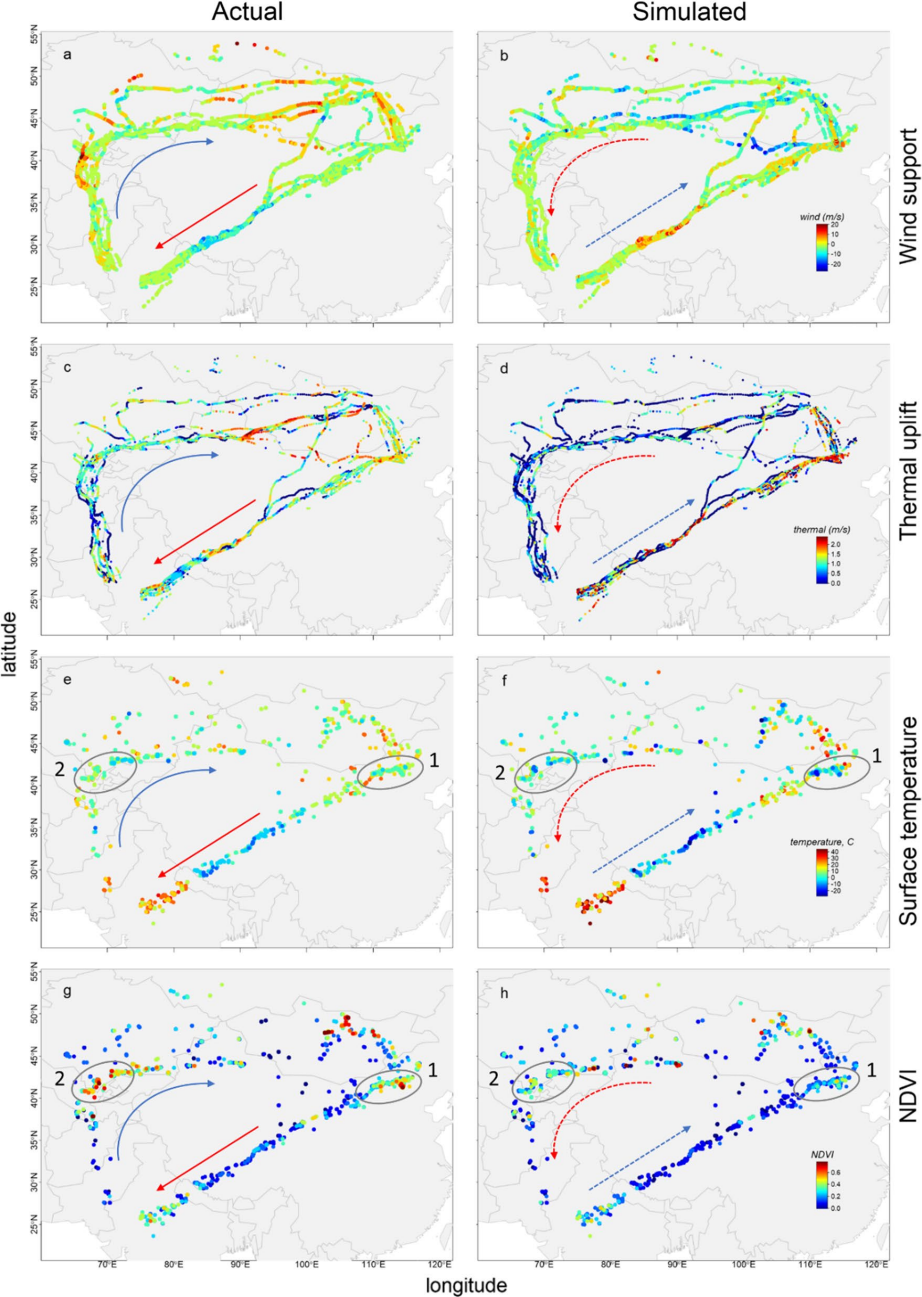
Actual migration (left) and simulated “reverse” migration path (right) of Demoiselle crane in relation to wind support (**a**, **b**), thermal uplift (**c**, **d**), temperature (**e**, **f**) and vegetation (NDVI; **g**, **h**) condition. The arrows indicate direction of (simulated) migration. Major staging sites where all birds stay during migration are highlighted by a numbered oval circle on the maps depicting on-ground conditions (**e**, **f**, **g**, **h**): 1- staging site in Inner Mongolia, 2-staging site near Aydar lake, south Kazakhstan

While comparisons of conditions aloft during actual and simulated migrations gave variable results, cranes would generally have encountered either poorer on-ground and/or aloft condition if they had migrated a simulated reverse route (Figs. 2, 3, Table 1). Wind support was better during actual outbound than simulated reverse migration. During inbound migration, wind support did not differ between actual and simulated reverse migration. Also thermal uplift during actual outbound migration was higher than during simulated reverse migration, it was similar during inbound migration. The surface temperature of actual outbound migration was three-fold higher than that of simulated reverse migration, while it did not differ between actual inbound and simulated reverse migration. For NDVI the reverse pattern was found, where NDVI was similar during actual and simulated reverse outbound migration, while, it was 86% higher for actual inbound compared to simulated reverse migration.

**Table 1:** Results of linear mixed effect models testing how conditions aloft (wind support and thermal uplift) and on-ground (temperature and NDVI) compare between reverse simulated migration and actual migratory conditions during both outbound and inbound migration

Dependent variable	Comparison (sample size, n)	Route comparison	Simulated—Actual			
			Estimate	Std. Error	*t*	*p *value
Wind support	sim. outb (n = 2779) – act. outb (n = 2315)	N-S	− 1.94	0.69	− 2.82	** < 0.05**
	sim. inb (n = 2279) – act. inb (n = 2818)	S–N	0.04	0.52	0.08	0.94
Thermal uplift	sim. outb (n = 1174) – act. outb (n = 2054)	N-S	− 0.38	0.07	− 5.26	** < 0.01**
	sim. inb (n = 1238) – act. inb (n = 2171)	S–N	0.17	0.08	2.05	0.07
Temperature	sim. outb (n = 18,831) – act. outb (n = 19,349)	N-S	− 8.26	2.85	− 2.90	** < 0.05**
	sim. inb (n = 19,349) – act. inb (n = 18,313)	S–N	− 0.02	2.73	− 0.01	0.99
NDVI	sim. outb (n = 18,416) – act. outb (n = 18,669)	N-S	− 0.03	0.02	− 1.73	0.10
	sim. inb (n = 18,201) – act. inb (n = 19,207)	S–N	− 0.12	0.03	− 3.68	** < 0.01**

Positive estimates indicate that the values are higher during reverse simulated migration, while negative estimates indicate they are higher during actual migration. The capital letter N denotes the northern route, while S denotes the southern route, crossing the Himalayas

### Comparison of conditions aloft during actual and simulated “time-shifted” migration

**Fig. 4:**
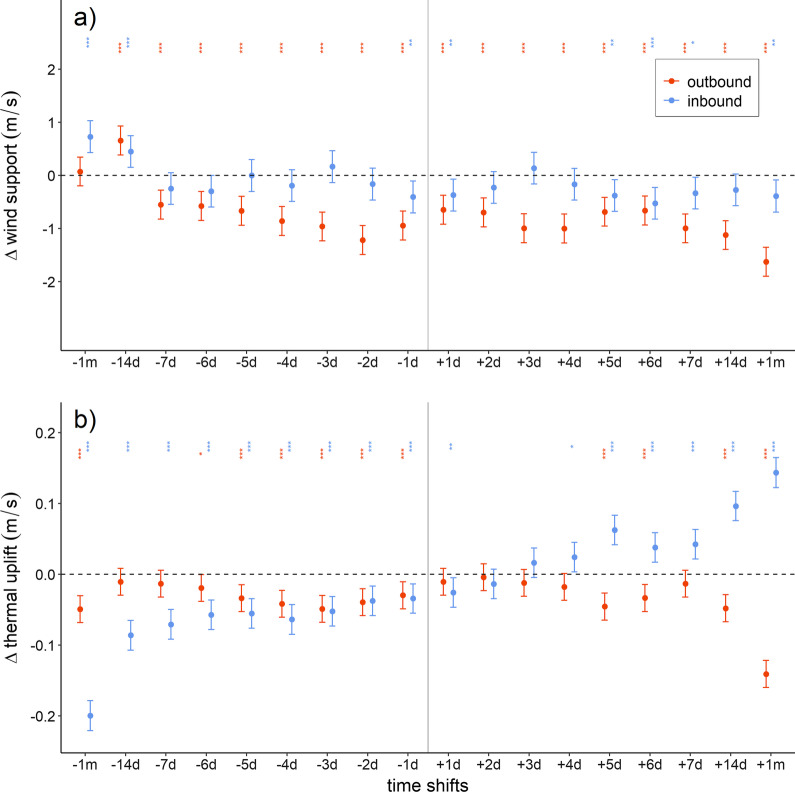
Difference in wind support (**a**) and thermal uplift (**b**) between simulated, time-shifted migration and actual migration of Demoiselle cranes. Outbound and inbound migrations are depicted using red and blue symbols, respectively. X-axes reflect the simulated time-shifts of migration ranging from ± 1 day to ± 1 month. All variables are represented as difference between time-shifted and actual conditions ± 95% family-wise confidence level. Positive values indicate better and negative values indicate poorer conditions aloft than on actual days of migration. The significance level of multiple-comparison Dunnett’s tests are indicated above each time-shift as *(*p* < 0.05), **(*p* < 0.01), and ***(*p* < 0.001). Horizontal dashed-line indicates the no-difference (i.e. same as actual) condition. Statistics and sample sizes are presented in Additional file 1

Cranes’ actual migratory conditions aloft were generally better than when time-shifted, with the exception of some cases of outbound thermal uplift (Fig. 4). During outbound migration, the cranes’ actual migration days had consistently better wind support than at other time-shifted moments (Fig. 4; Additional file 1: Table S2). During inbound migration, cranes also selected actual migration days with higher wind support compared to one-day-earlier and later, and also five to seven days and a month later time-shifts (i.e., -1d, + 1d, + 5d to + 7d, and + 1 m), even though the majority of time-shifted migrations had positive (i.e., more) wind support (Fig. 4; Additional file 1: Table S3). Similarly, the cranes’ actual migration days had better thermal uplift than most of the time-shifts, notably when compared with earlier time-shifts. During outbound migration, the cranes’ actual migration days had higher thermal uplift than during the majority of the time-shifts. However, conditions would have been similar if they had migrated one to two weeks before or one to three days later (Fig. 4; Additional file 1: Table S4). During inbound migration, actual migration days also had higher thermal uplift than all earlier time-shifts as well as the one-day-later time-shift. It should be noted though that thermal uplift is expected to increase day by day in spring, following the gradual increase in ambient temperature. Hence, cranes would have had even higher thermal uplift if they had migrated four days later (Fig. 4; Additional file 1: Table S5).

## Discussion

Demoiselle cranes apparently undertake their loop migration to exploit both favourable stopover, on-ground, as well as aloft conditions. At refuelling sites NDVI is an important correlate of food supply for migrants [1, 46–49]. By migrating via a more northerly inbound route, rather than using the trans-Himalayan trajectory used during outbound migration, the cranes experienced 86% more vegetation cover than if they had engaged in a straight return migration across the Himalayas (i.e. a simulated reverse migration). During outbound migration, by crossing the Himalayas rather than avoiding them using a northern loop, the birds experienced better wind support, thermal uplift and higher surface temperatures (see Fig. 2). In the final stages of the preparation of this manuscript we became aware of the work of Mi et al. [50] who similarly to us investigated the environmental factors underlying the distinguished loop migration in Demoiselle cranes. While using different data set and analyses their conclusions importantly ovelap in that both aloft and on-ground conditions appear to be key on explaining the differential migration routes for inbound and outbound migration. It has been suggested that Trans-Himalayan spring migration might be a harsh undertaking for waterfowl [51]. Notably in spring, on-ground conditions on the Tibetan Plateau often involving sub-zero temperatures, potentially impacting food availability, resulting in a true migration barrier for birds such as cranes that require en route replenishment of fuel stores [28, 52–54]. Despite this, many raptors and waterfowl nevertheless do cross the Himalayas during spring migration, following a similar route as their trans-Himalayan outbound migration [55–57]. These differences between cranes on the one hand and raptors and waterfowl on the other, may be explained by flight mode and feeding ecology. Migration is most costly for migrants using powered, flapping flight [1, 58] such as waterfowl. Detours around barriers may thus be more costly for such birds than for soaring migrants or those using a mixture of soaring and powered flight, such as cranes. Next, raptors which mainly prey on mammals and birds may be less impacted by low vegetation cover. Also herbivores that are not only relying on above ground vegetation but also forage on tubers and stolons, such as many herbivorous waterfowl, may be less sensitive to low above ground vegetation cover. In contrast, Demoiselle cranes mainly feed on items that are available only when the productive season has already somewhat progressed (cultivated crops and their remains, other above ground vegetation) [28, 59]. Thus, the loop migration of Demoiselle cranes is probably best explained in terms of their specific dietary needs and relatively low flight costs while being able to soar.

Additional support for the importance of aloft conditions shaping migration strategies comes from the comparisons of simulated time-shifted migration with actual migration data showing that they tended to undertake migratory flights on days with more favourable tailwind and thermal uplift (see Fig. 4). In addition, we found that cranes undertook the majority of migratory flights during midday when intensity of thermal uplift is at its highest (see Additional file 1: Fig. S2). That partially soaring migrants actively select days of favourable thermal conditions has not been investigated previously, although Common cranes (*Grus grus*) occasionally undertake soaring migration flights [26, 27]. To summarise, while both on-ground and aloft conditions are critical in explaining the loop migration of Demoiselle cranes, condition aloft may impact flight costs and importantly determine the actual days on which migrations take place.

The energetic consequences of variations in conditions aloft for migrants have been shown to determine variations in migratory routes and long foraging flights [60, 61] and are importantly thought to have shaped the evolution of migratory routes [1]. Also in this study, conditions aloft during actual migration trajectory was more favourable during outbound migration. However, it should be considered that simulated reverse migration probably underestimates thermal uplift and wind support, because it does not consider that birds actually chose favourable days for migratory flight (cf. Fig. 4), conditions aloft during reverse simulated migration might thus have been slightly better than depicted in Figs. 2 and 3. Moreover, it should be considered that aside from the here used calculation of wind-assistance, many other alternatives exist that could yield different outcomes and may (sometimes) be more appropriate depending on how Demoiselle cranes respond to flying in a moving medium route [62]. Another caveat that should be mentioned is that during our simulated reverse migration not only the aloft but also the stop-over conditions could potentially be underestimated, because the location of the stopover is fixed to the locations used by the Demoiselle cranes during the actual migration. However, inspection of the (NDVI) maps showed that if the Demoiselle cranes would have chosen to fly the routes at other times of the year, the locations where they stopped during actual migration would likely be the most suitable and productive at those other moments in the year.

Avian loop migrations are thought to be a very common migration phenomena, notably in the North-American and the Afro-Palearctic flyways [11, 19]. However, the underlying causes for loop migrations have rarely been addressed, with the exception of studies by Klaassen et al. [9] for Marsh harrier migration in the Afro-Palearctic flyway and Sorte et al. [8] for terrestrial birds in western North America. Marsh harriers migrating between southern Sweden and western Africa, appeared to migrate in a relatively narrow, clockwise loop between southern Europe and western Africa [9]. The authors of that study tentatively concluded that general wind condition rather than foraging condition explained this loop migration. During inbound migration, many songbirds in western North America tend to use a more westerly and lower elevation route than during outbound migration, for which loop Sorte et al. [8] found differences in ecological productivity at the respective times of year to be responsible. Aside from these two studies that dealt with rather small loops, there have been other loop migrations for which mechanisms have been suggested, yet, not researched. For probably the biggest avian loop migration that has been documented to date, i.e. for Bar-tailed godwits (*Limosa lapponica*) in the Pacific, Gill Jr et al. [63] hypothesized that low food availability and high energetic demands upon arrival at the breeding grounds required a penultimate top-up stop in relatively close proximity to those breeding grounds, driving this loop. Thus, our study in conjunction with existing studies and hypotheses [5, 8, 9, 11, 19, 63] on the underlying causes of loop migration show that the underlying factors determining loop migrations can vary across different geographical regions and taxonomic groups. Yet, in all cases the better understanding of loop migrations elucidates the constraints acting upon migrants and how these may vary between outbound and inbound migration, with critical implications for the conservation of migrants and the habitats on which they rely.

## Conclusions

Although wind support and thermal uplifts are often crucial for successful migration, it was also on ground conditions in addition to these atmospheric factors that helped explain the remarkable loop migration in which Demoiselle cranes in East Asia engage. Our analyses notably suggested that better foraging conditions (i.e. higher NDVI) along the inbound route was explaining their preference to fly around the Himalayas rather than across. We therefore conclude that seasonal variations in on-ground and aloft environmental conditions importantly drive their choice of migration route. We consider that the here employed approach not only assist in understanding the trajectories of loop migrations but potentially also detour migrations. This approach and additional knowledge on the drivers for choice of migration trajectories facilitates predicting migration decisions and drafting mitigation strategies for global change effects on animal migrations.

## Supplementary Information

**Additional file 1.** **Figure S1.** Illustration outlining reverse migration simulation. **Table S1.** Summary of environmental conditions during outbound and inbound migration of Demoiselle crane. **Table S2.** Differences in wind support of time-shifted outbound migration from actual migration. **Table S3.** Differences in wind support of time-shifted inbound migration from actual migration. **Table S4.** Differences in thermal uplift of time-shifted outbound migration from actual migration. **Table S5.** Differences in thermal uplift of time-shifted inbound migration from actual migration. **Figure S2.** Hourly distribution of wind support (**a**) and thermal uplift (**c**) flight height (**b**) and number of in-flight fix (**d**) during outbound and inbound migration.

## Data Availability

The tracking data analysed during the current study is available on Movebank, under study names: LifeTrack Mongolia Demoiselle cranes and Demoiselle Crane High Resolution Mongolia. The annotated datasets analysed in this study is available on Dryad (https://doi.org/10.5061/dryad.cnp5hqc1r).
