# Supplementary material for: Differences in on-ground and aloft conditions explain seasonally different migration paths in Demoiselle crane
Source: Mov Ecol. 2022 Jan 31;10:4. doi: 10.1186/s40462-022-00302-z (PMC8805327; doi:10.1186/s40462-022-00302-z)
Supplement: Supplementary file 1 — Additional file 1. Figure S1. Illustration outlining reverse migration simulation. Table S1. Summary of environmental conditions during outbound and inbound migration of Demoiselle crane. Table S2. Differences in wind support of time-shifted outbound migration from actual migration. Table S3. Differences in wind support of time-shifted inbound migration from actual migration. Table S4. Differences in thermal uplift of time-shifted outbound migration from actual migration. Table S5. Differences in thermal uplift of time-shifted inbound migration from actual migration. Figure S2. Hourly distribution of wind support (a) and thermal uplift (c) flight height (b) and number of in-flight fix (d) during outbound and inbound migration. [file 40462_2022_302_MOESM1_ESM.pdf]

ADDITIONAL FILE

Methods

Simulation of reverse migration

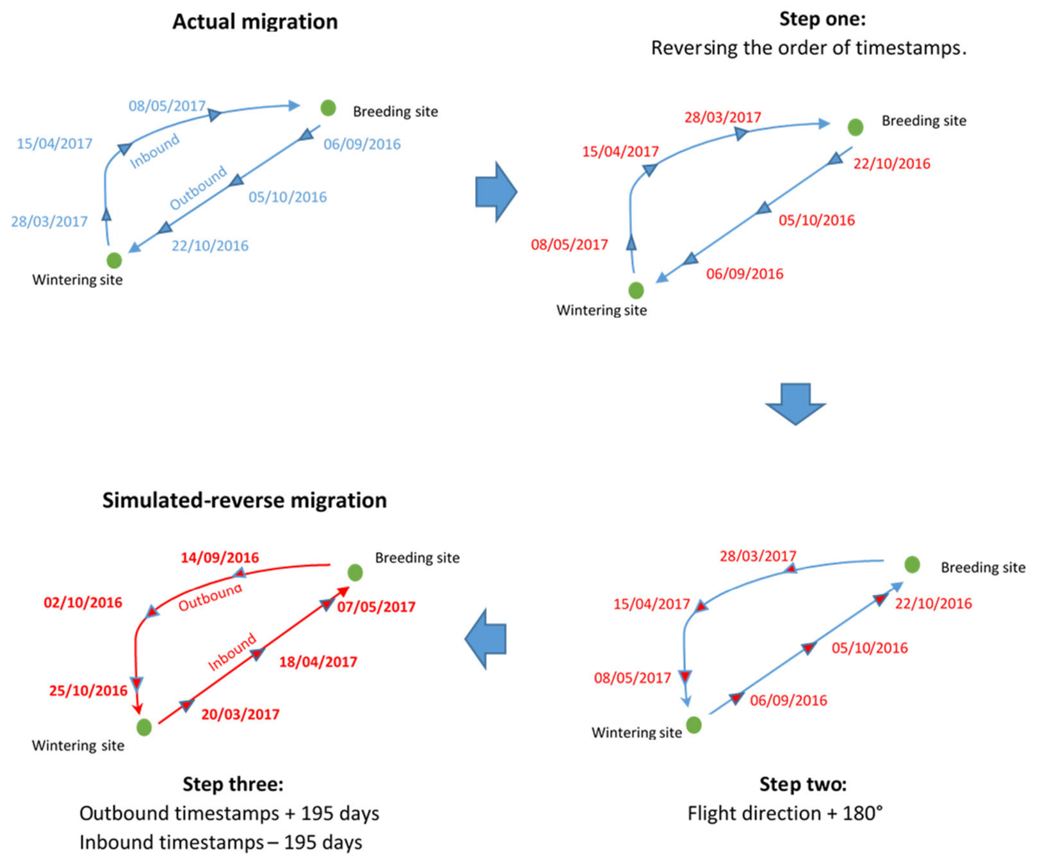

Figure S1. Illustration outlining reverse migration simulation. Top left diagram represents an actual migration. In the first step the timestamp variable is reversed for each migratory track using the rev function in base R (top right diagram). Next, we reversed the flight direction by adding 180° (bottom right diagram). Finally, we added 195 days on the reversed timestamps of the outbound migration and subtracted 195 days from the reversed timestamp of the inbound migration.

Result

Migratory route

Cranes started their outbound migration in early September (median 5th September), heading southeast over one to two days to their first staging sites in Inner Mongolia except for one individual, which flew directly to the Tibetan Plateau crossing the Gobi Desert (Fig. 1). By the end of September, after spending 12-26 days (19±5, n=16; hereafter mean ±SD is provided in parenthesis) at these staging sites, they initiated their longest migratory leg (4,098 km, n=16) by flying straight across the Himalayan Mountains, heading southwest to their first potential wintering ground in an agricultural area in Rajasthan province, north-western India. This journey took 14-40 days (24±8, n=16) interspersed with short stops lasting a maximum of three days. The first part of this leg, until the birds started crossing the Himalayan Mountains, took 10-15 days (13±2, n=16) with short stops along rivers and streams in between Medu-Kun and Gar Kangri Mountains on the Tibetan plateau. After crossing the Himalayas, six individuals staged for 6-76 days (33±25, n=6) at different agricultural sites in Uttar Pradesh and Madhya Pradesh provinces before reaching Rajasthan. Two individuals did not stop at Rajasthan but directly flew on in south-westerly direction to coastal Gujarat province, India, which is a second major wintering site for Demoiselle cranes. Thirteen cranes, of which the transmitters were still transmitting at the time, spent 6-37 days (21±11, n=16) at Rajasthan, after which they moved on to Gujarat. In Gujarat, the cranes stayed for 107-149 days (123±13, n=13). Thus, including times at stopover, outbound migration took 29-72 days (48±12, n=15), to cover a great-circle-route distance of 4,896±223 km (n=15), excluding the distance between the first and second wintering ground of an additional 500 km.

Inbound, spring migration started by the end of March (median departure 25th March) when the cranes flew north to Aydar Lake in Uzbekistan and south Kazakhstan, which they reached 3-18 days later (7±6, n=12). The birds stayed in this area for 5-25 days (14±10, n=12). By mid-April the cranes moved on via different routes to their breeding ground. Three cranes took a straight route to their

breeding site in Northeast Mongolia, while others took different routes, one through Sayan Mountains and other three via the foothills of the Tian Shan Mountains. These journeys took 8-55 days (30±20, n=9) with up to 25 days of staging at a variety of sites along the route. Thus, including the times at stopover, duration of inbound migration totalled 35-66 days (54±14, n=9), covering a great-circle-route distance of 6,034±518 km (n=9).

Environmental conditions during migration

Environmental conditions varied greatly during both outbound and inbound migrations, and both aloft (wind support =-21.1 – 24.1 m/s (0.2±4.3, n=5,133); thermal uplift =0.04 – 2.2 m/s (1.1±0.4, n=5,133)) and on the ground (ambient temperature =-12.6 – 36.7 °C (15.2±8.2, n=38,162); NDVI=0.0 – 0.8 (0.27±0.16, n=38,162)) (table S1 additional file). During the first part of outbound migration to Inner Mongolia, the birds were on average experiencing a tailwind, facing strongest headwinds when crossing the Tibetan plateau before crossing the Himalayan Mountains (Fig. 3a). During inbound migration flight, cranes experienced an average wind support of 0.76±3.92 m/s (table S1), with average wind support differing significantly between seasons (t-test=16.7, df=4,663, $p<0.001$). Thermal uplift did not differ between the migratory seasons (t-test=-0.09, df=4,083, $p>0.05$), and cranes encountered similar thermal uplifts (1.1±0.4 m/s) throughout most of their journey.

When on-ground during outbound migration, cranes experienced relatively warm temperatures until they reached the Tibetan Plateau, where they faced considerably colder temperatures, often reaching sub-zero levels (Fig. 3e). The surface temperature was on average 15.4±8.6 °C with a lowest temperature of -12.6 °C on the Tibetan Plateau and a highest temperature of 35.4 °C in Rajasthan, India. During inbound migration, the average surface temperature was 14.2±7.8 °C and slightly lower than during outbound migration (t-test=-14.07, df=37,996, $p<0.001$). The highest temperature of 36.7 °C was recorded in Pakistan, while the lowest temperature of -10.4 °C was recorded in southeast Kazakhstan (Fig. 3c). NDVI was generally highest at the major stopover sites, i.e. in Inner

Mongolia during outbound migration and the border between Uzbekistan and South Kazakhstan during inbound migration (Fig. 3g). During outbound migration, the average NDVI value at stopover sites was 0.28±0.1, with the highest (0.81) and lowest (0.00) NDVI values recorded in Nepal and on the Tibetan Plateau, respectively. During inbound migration, the average NDVI was slightly higher at 0.31±0.2 (t-test=11.5, df=36,468, p<0.001), with the highest (0.81) and lowest (0.00) NDVI values recorded in the Surxondaryo Region, Uzbekistan, and in the Gobi desert in Inner Mongolia, respectively (table S1).

Table S1. Summary of environmental conditions during outbound and inbound migration of Demoiselle crane. Values are estimated from the weather data annotated to all aloft and on-ground fixes. P-values of t-tests are provided in bold if significant (p<0.05).

Variables	Migratory route	Mean	Median	SD	N	Min	Max	p-value
Wind support, m/s	Outbound	-0.98	-0.50	4.4	2,315	-21.1	11.9	**< 0.001**
	Inbound	0.99	0.76	3.9	2,818	-11.4	24.1	
Thermal uplift, m/s	Outbound	1.08	1.10	0.3	2,315	0.1	2.0	0.92
	Inbound	1.08	1.09	0.4	2,818	0.0	2.2	
Temperature, °C	Outbound	15.40	16.20	8.6	19,349	-12.6	35.4	**< 0.001**
	Inbound	14.20	14.30	7.8	18,813	-10.4	36.7	
NDVI	Outbound	0.29	0.28	0.2	19,349	-0.2	0.8	**< 0.001**
	Inbound	0.31	0.27	0.2	18,813	0.0	0.8	

Table S2. Differences in wind support of time-shifted outbound migration from actual migration. Estimates are the result of multiple comparison with Dunnett contrast on the linear mixed effect model. Formula: glht (lmer (Wind support ~ time shifts + (1 | Individual id / fix id), data = outbound), linfct = mcp (shifts = "Dunnett") , alternative="two.sided"). R^2m and R^2c stand for marginal and conditional R^2, respectively.

Dependent variable	Time shifts	n. of fix	Estimate	Std. Error	*z*	*p*	R^2m	R^2c
Wind support outbound	-1m	2,469	0.07	0.09	0.75	0.99	0.01	0.51
	-14d	2,469	0.65	0.09	6.92	**< 0.001**		
	-7d	2,469	-0.55	0.09	-5.86	**< 0.001**		
	-6d	2,469	-0.58	0.09	-6.12	**< 0.001**		
	-5d	2,469	-0.67	0.09	-7.06	**< 0.001**		
	-4d	2,469	-0.86	0.09	-9.10	**< 0.001**		
	-3d	2,469	-0.96	0.09	-10.20	**< 0.001**		
	-2d	2,469	-1.22	0.09	-12.90	**< 0.001**		
	-1d	2,469	-0.94	0.09	-10.00	**< 0.001**		
	+1d	2,469	-0.65	0.09	-6.86	**< 0.001**		
	+2d	2,469	-0.70	0.09	-7.40	**< 0.001**		
	+3d	2,469	-1.00	0.09	-10.56	**< 0.001**		
	+4d	2,469	-1.00	0.09	-10.61	**< 0.001**		
	+5d	2,469	-0.69	0.09	-7.26	**< 0.001**		
	+6d	2,469	-0.66	0.09	-7.03	**< 0.001**		
	+7d	2,469	-1.00	0.09	-10.57	**< 0.001**		
	+14d	2,469	-1.12	0.09	-11.90	**< 0.001**		
	+1m	2,470	-1.63	0.09	-17.24	**< 0.001**		
	actual	2,469						

Table S3. Differences in wind support of time-shifted inbound migration from actual migration. Estimates are the result of multiple comparison with Dunnett contrast on the linear mixed effect model. Formula: glht (lmer (Wind support ~ time shifts + (1 | Individual id / fix id), data = inbound), linfct = mcp (shifts = "Dunnett") , alternative="two.sided"). R^2m and R^2c stand for marginal and conditional R^2, respectively.

Dependent variable	Time shifts	n. of fix	Estimate	Std. Error	*z*	*p*	R^2m	R^2c
Wind support inbound	-1m	2,993	0.73	0.10	7.01	**< 0.001**	0.00	0.21
	-14d	2,979	0.45	0.10	4.31	**< 0.001**		
	-7d	2,982	-0.25	0.10	-2.40	0.17		
	-6d	2,982	-0.30	0.10	-2.88	0.05		
	-5d	2,982	0.00	0.10	-0.04	1.00		
	-4d	2,982	-0.19	0.10	-1.87	0.46		
	-3d	2,982	0.16	0.10	1.57	0.69		
	-2d	2,982	-0.17	0.10	-1.59	0.68		
	-1d	2,982	-0.41	0.10	-3.92	**< 0.01**		
	+1d	2,982	-0.37	0.10	-3.58	**< 0.01**		
	+2d	2,978	-0.23	0.10	-2.20	0.26		
	+3d	2,982	0.14	0.10	1.30	0.88		
	+4d	2,980	-0.17	0.10	-1.61	0.67		
	+5d	2,980	-0.38	0.10	-3.67	**< 0.01**		
	+6d	2,982	-0.53	0.10	-5.07	**< 0.001**		
	+7d	2,982	-0.33	0.10	-3.21	**< 0.05**		
	+14d	2,981	-0.28	0.10	-2.65	0.09		
	+1m	2,762	-0.39	0.11	-3.68	**< 0.01**		
	actual	2,982						

Table S4. Differences in thermal uplift of time-shifted outbound migration from actual migration. Estimates are the result of multiple comparison with Dunnett contrast on the linear mixed effect model. Formula: glht (lmer (thermal ~ time shifts + (1 | Individual id / fix id), data = outbound), linfct = mcp (shifts = "Dunnett") , alternative="two.sided"). R^2m and R^2c stand for marginal and conditional R^2, respectively.

Dependent variable	Time shifts	n. of fix	Estimate	Std. Error	*z*	*p*	R^2m	R^2c
Thermal uplift outbound	-1m	2,553	-0.05	0.01	-7.51	**<0.001**	0.005	0.69
	-14d	2,621	-0.01	0.01	-1.63	0.65		
	-7d	2,586	-0.01	0.01	-2.05	0.34		
	-6d	2,603	-0.02	0.01	-2.94	**<0.05**		
	-5d	2,579	-0.03	0.01	-5.13	**<0.001**		
	-4d	2,620	-0.04	0.01	-6.35	**<0.001**		
	-3d	2,618	-0.05	0.01	-7.42	**<0.001**		
	-2d	2,565	-0.04	0.01	-6.01	**<0.001**		
	-1d	2,544	-0.03	0.01	-4.53	**<0.001**		
	+1d	2,508	-0.01	0.01	-1.64	0.65		
	+2d	2,642	0.00	0.01	-0.65	1.00		
	+3d	2,580	-0.01	0.01	-1.89	0.45		
	+4d	2,616	-0.02	0.01	-2.71	0.08		
	+5d	2,590	-0.05	0.01	-6.95	**<0.001**		
	+6d	2,614	-0.03	0.01	-5.09	**<0.001**		
	+7d	2,645	-0.01	0.01	-2.03	0.36		
	+14d	2,620	-0.05	0.01	-7.26	**<0.001**		
	+1m	2,484	-0.14	0.01	-21.09	**<0.001**		
	actual	2,632						

Table S5. Differences in thermal uplift of time-shifted inbound migration from actual migration. Estimates are the result of multiple comparison with Dunnett contrast on the linear mixed effect model. Formula: glht (lmer (thermal ~ time shifts + (1 | Individual id / fix id), data = inbound), linfct = mcp (shifts = "Dunnett") , alternative="two.sided"). R^2m and R^2c stand for marginal and conditional R^2, respectively.

Dependent variable	Time shifts	n. of fix	Estimate	Std. Error	*z*	*p*	R^2m	R^2c
Thermal uplift inbound	-1m	2,116	-0.20	0.01	-27.05	**< 0.001**	0.02	0.74
	-14d	2,238	-0.09	0.01	-11.89	**< 0.001**		
	-7d	2,236	-0.07	0.01	-9.75	**< 0.001**		
	-6d	2,240	-0.06	0.01	-7.91	**< 0.001**		
	-5d	2,281	-0.06	0.01	-7.69	**< 0.001**		
	-4d	2,230	-0.06	0.01	-8.80	**< 0.001**		
	-3d	2,255	-0.05	0.01	-7.24	**< 0.001**		
	-2d	2,254	-0.04	0.01	-5.20	**< 0.001**		
	-1d	2,275	-0.03	0.01	-4.77	**< 0.001**		
	+1d	2,266	-0.03	0.01	-3.60	**<0.01**		
	+2d	2,281	-0.01	0.01	-1.92	0.43		
	+3d	2,273	0.02	0.01	2.23	0.24		
	+4d	2,283	0.02	0.01	3.33	< 0.05		
	+5d	2,308	0.06	0.01	8.64	**< 0.001**		
	+6d	2,347	0.04	0.01	5.24	**< 0.001**		
	+7d	2,281	0.04	0.01	5.87	**< 0.001**		
	+14d	2,319	0.10	0.01	13.37	**< 0.001**		
	+1m	2,206	0.14	0.01	19.59	**< 0.001**		
	actual	2,268						

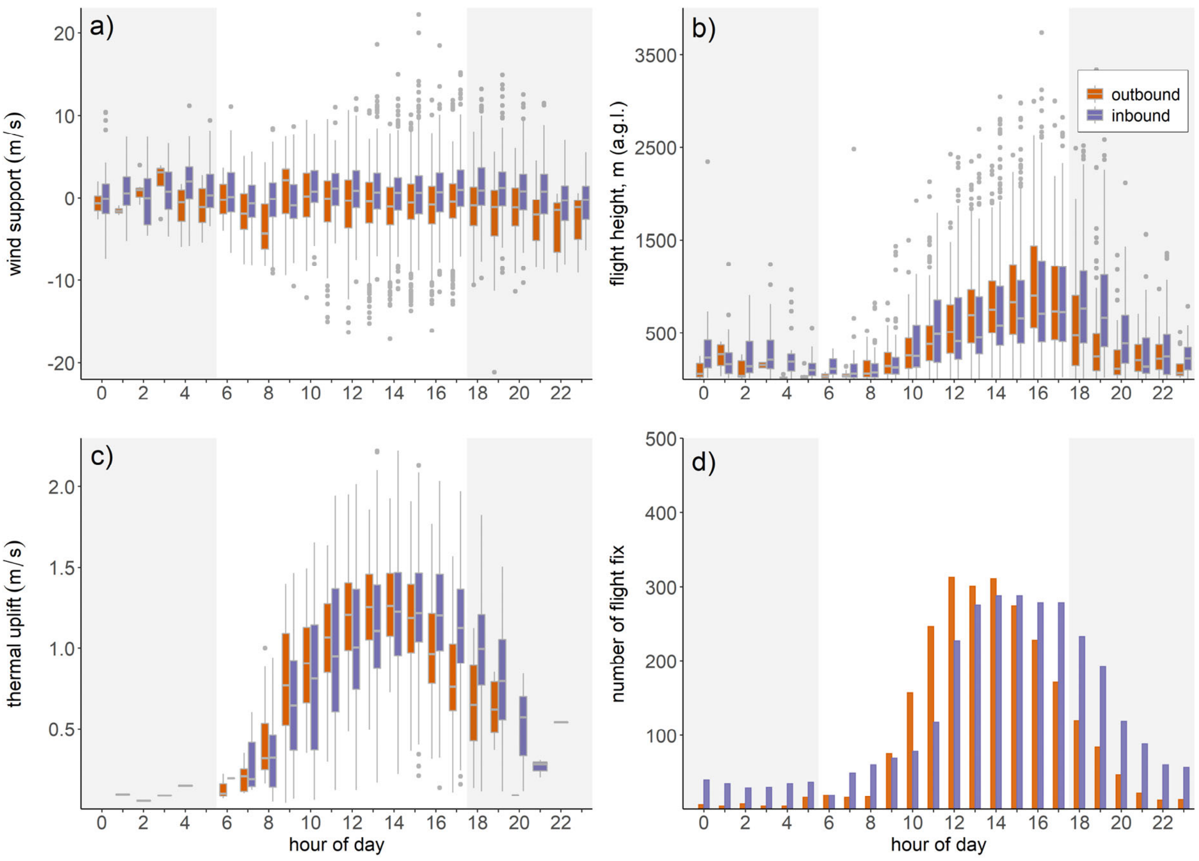

Figure S2. Hourly distribution of wind support (a) and thermal uplift (c) flight height (b) and number of in-flight fix (d) during outbound and inbound migration.
